# Systematic synthesis of community-based rehabilitation (CBR) project evaluation reports for evidence-based policy: a proof-of-concept study

**DOI:** 10.1186/1472-698X-8-3

**Published:** 2008-03-06

**Authors:** Pim Kuipers, Sheila Wirz, Sally Hartley

**Affiliations:** 1 CONROD, University of Queensland and Centre for Remote Health (a joint centre of Flinders University and Charles Darwin University). Centre for Remote Health, P.O. Box 4066 Alice Springs NT 0870, Australia; 2 Centre for International Health and Development, University College London, UK; 3 Faculty of Health, University of East Anglia, UK

## Abstract

**Background:**

This paper presents the methodology and findings from a proof-of-concept study undertaken to explore the viability of conducting a systematic, largely qualitative synthesis of evaluation reports emanating from Community Based Rehabilitation (CBR) projects in developing countries.

**Methods:**

Computer assisted thematic qualitative analysis was conducted on recommendation sections from 37 evaluation reports, arising from 36 disability and development projects in 22 countries. Quantitative overviews and qualitative summaries of the data were developed.

**Results:**

The methodology was found to be feasible and productive. Fifty-one themes were identified and the most important ones of these are presented to illustrate the significance of the method. The relative priorities of these themes indicated that "management" issues were the primary areas in which recommendations were made. Further analysis of themes reflected the emphasis evaluators placed on the need for enhanced management, organisational, personnel and administrative infrastructure in CBR projects. Evaluators consistently recommended that CBR projects should be more connected and collaborative at governmental, organisational, political and community levels. The synthesis also noted that evaluators questioned the emphasis in CBR on project expansion and income generation.

**Conclusion:**

The application of the synthesis methodology utilised in this proof-of-concept study was found to be potentially very beneficial for future research in CBR, and indeed in any area within health services or international development in which evaluation reports rather than formal "research evidence" is the primary source material. The proof-of-concept study identified a number of limitations which are outlined. Based on the conclusions of 37 evaluation reports, future policy frameworks and implementation strategies in CBR should include a stronger emphasis on technical, organisational, administrative and personnel aspects of management and strategic leadership.

## Background

To date, research and publication within the area of disability and development, and specifically that pertaining to community based rehabilitation (CBR) service delivery, has primarily been descriptive in nature. While this has led to considerable advances in understanding processes and issues, it has not resulted in clear policy, evidence of efficacy, or practice directives. The recent review of CBR, initiated by the W.H.O. [1], identified that, *Governments want evidence-based practices, so CBR programmes must be ready to provide evidence *(p15). More specifically, they charged researchers and NGOs to *work on evaluation and research on evidence-based practices *(p20), in order to contribute to planning, policy making and to assist with decision-making for the scaling-up of CBR globally. Unfortunately, with the exception of a recent detailed review [2], and some specific reviews [3], there are few studies in CBR which might be used to contribute to such an evidence base within traditional approaches to determining evidence.

However, the field of evidence-based practice is broadening, and in recent years there has been considerable debate regarding the nature of evidence, potential sources of evidence and the suitability of different types of evidence to inform healthcare [4]. Emerging approaches to the synthesis of evidence are now moving beyond traditional published (or black) literature, and even non peer-reviewed (or grey) literature, and are expanding to include other forms of data, rarely utilized in traditional research [5]. Mays, Pope & Popay [6] have challenged researchers to develop appropriate methodologies to accommodate and maximize these wide ranging and disparate types of evidence.

In light of these developments in the health field, we proposed a pragmatic methodological framework for identifying evidence in CBR [7]. We suggested that a highly collaborative and detailed qualitative synthesis of evaluation reports would constitute a substantial contribution to evidence in this area. We noted that such a process would result in general evidence for policy, rather than specific evidence for CBR interventions, and anticipated that the resultant evidence would be representative of project-level concerns in developing countries.

We recognised that to establish recognition of the features of such an innovative methodology (including a strongly collaborative process, the use of evaluation reports as data and the use of qualitative analysis), it would be important to conduct preliminary studies. Since the imperative for collaborative, client-led research is well established in this area, consistent with the philosophy of inclusive development [8], and seen as a key principle of practice in qualitative systematic review [9], we reasoned that this feature did not require further justification. In the current proof-of-concept study, we sought to explore both the viability of analysing evaluation reports as a data source, and the suitability of conducting qualitative thematic analysis as a methodology for this data.

In order to do so, some issues had first to be clarified. Key among these was concern over the appropriateness, and use of evaluation reports to inform evidence. Evaluations usually pertain to individual projects and aggregating content across reports may conflict with their authors' intent. However, we noted that each of these unique evaluation reports is also partly descriptive of a larger underlying phenomenon [6] namely, day-to-day CBR service delivery. As such, evaluation reports are potential elements of a representative composite data set describing CBR service delivery, which can be synthesised and scrutinised with the appropriate (predominantly qualitative) methodology.

A related issue was the concern that the aggregation of multiple instances of such data could result in amorphous or diluted constructs from which few meaningful conclusions could be derived. In recognition of this concern, we undertook the current proof-of-concept study to explore whether such a synthesis would lead to ill-defined outputs, or whether the proposed strategy of synthesis of evaluation reports would actually result in robust, discernable and meaningful themes.

Selecting a component from each report to test the methodology proved challenging, as the reports were heterogeneous by nature. We reasoned that the most practical and manageable section to analyse using our proposed method would be the "recommendations", since nearly all reports include a recommendations section. Focusing our synthesis on these sections would allow us to apply and test procedural aspects of the methodology in a relatively short space of time on the most conceptually rich and meaningful part of each evaluation report. Further, it would allow us to consider strengths and limitations of the methodology (acknowledging of course a possible bias towards focusing on the shortcomings of projects). With regard to the concerns noted above, we proposed that if such a synthesis were to demonstrate the viability of the methodology, it should be able to be conducted relatively straightforwardly, and result in meaningful and discernable themes, which are grounded in the data.

## Methods

In keeping with similar studies [10], source materials for this study were sought from formal and personal sources. A total pool of 58 potential evaluation reports was identified, which comprised all of the CBR-related evaluation reports held in 'Source', as well as those in the personal libraries/resources of the three authors. (Source, based at the Centre for International Health and Development, University College London, is an international information support centre, designed to strengthen the management, use and impact of information on health and disability [11].)

Selection criteria for inclusion were:

a. a written report of a formal evaluation, conducted within a defined time period,

b. written in English,

c. specifically pertaining to disability related work,

d. conducted entirely in a developing country,

e. between 1996 and 2006, and

f. excluding final evaluation reports, or reports of projects that were about to cease.

Recognising that CBR is far from uniform [12], the post-1996 time frame was chosen since it was considered that this would to some extent maximize uniformity of concepts, coming at least two years after the widely adopted 1994 Joint Position Paper [13]. In this initial proof-of-concept study, no judgments were made regarding quality, process, focus, project type, evaluator, or region of the project evaluation reports. Thirty-seven (of the original 58) reports meeting the above criteria were included [see Additional file 1]. They comprised 13 reports emanating from projects in Africa, 12 from South-East Asia, 6 from South Asia, and 6 from other regions (Middle East, Americas, and Eastern Europe).

"Recommendations" sections of these 37 CBR-related evaluation reports were extracted and converted to electronic text format. The process of retrieval of recommendations varied depending on the available format and layout of each report. In some instances it involved the location of specific recommendations from throughout the body of the report; in most cases it comprised a more straightforward electronic cutting-and-pasting of a recommendations section into a text file. All recommendations from all reports were included and coded as they appeared in the evaluation report.

### Coding and analysis

Thematic analysis and synthesis of all recommendations in the 37 reports was undertaken to identify salient themes and patterns [14]. Initially, all recommendations were read by the first author. They were then entered into and organised using N-Vivo qualitative data analysis software [15] which facilitated the process of coding (categorisation and labelling). Themes and corresponding codes were identified as they emerged in progressive readings of the recommendations. That is, through a process of categorisation and comparison, recurring themes were noted within the text and assigned to a "node" with similar data (data-driven coding). This process resulted in 51 nodes (or themes) derived from the 37 evaluation report recommendation sections; each node was then printed and reviewed.

In order to ensure reliability of coding and categorisation, a two-phase process was undertaken. First, as a colleague check, an experienced researcher and CBR practitioner, not involved in the current project coded paragraphs from 10 randomly selected reports, independently of the first author and the two coding systems were compared. Fifty six percent of coded statements were found to be common to both. Further examination revealed that statements which were not commonly coded, differed on minor matters of interpretation, which did not affect the themes of the study (eg using the node "collaborating organisation" rather than "NGO"). Next, two authors and the CBR practitioner independently reviewed the summaries of all 51 nodes. Each developed a framework for organising the 51 nodes into conceptual categories. Through a process of comparison and refinement, they agreed by consensus on the final conceptual category structure of the nodes depicted in Table 1.

**Table 1:** Frequency of coding for nodes and categories of nodes (themes)

**Node (Theme)**	**No. of evaluation reports which include theme (N = 37)**	**Extent of Data**	**Category of node or theme**	**No. of evaluation reports which include category (N = 37)**
Funding, Finances	28	* * *	**MANAGEMENT ISSUES**	**36**
Collaborating Organisation	28	* * *		
Management Issues	23	* *		
Government – Links Agencies Depts.	23	* *		
NGO INGO	19	* *		
Documentation	18	*		
Organisational Development	18	*		
Administrative	14	*		
PR and publicity	10	*		
Infrastructure	1			

Expansion	23	* *	**LEADERSHIP AND FUTURE DIRECTION ISSUES**	**35**
Planning	15	*		
Policy	15	*		
Sustainability	8			
Decentralisation	7			
Deinstitutionalisation	3			

Community Involvement	21	* *	**IMPROVING CBR AT INDIVIDUAL/COMMUNITY LEVEL**	**33**
DPOs self help groups	18	*		
Participation in project	17	*		
Volunteers	16	*		
Family Involvement	15	*		
Attitudes to people with disability	11	*		
Grass-Roots Orientation	11	*		
Village Committees	5			
Cultural Relevance	3			
Sexuality	3			

Training	30	* * *	**PERSONNEL MANAGEMENT ISSUES**	**29**
CBR worker LS, Role, Recruit	16	*		
Staff Development	13	*		
Work Process, Teams	8			

Research	11	*	**IMPROVING CBR MODEL/APPROACH**	**29**
Empowerment	9			
Gender	8			
Advocacy	8			
Holistic Approach	7			
Human Rights	5			
Negatives, Description of Problem	3			
Ethics Confidentiality	3			

Education – integrated	18	*	**PROJECT SERVICE PROVISION ISSUES**	**28**
Income-Generation Vocational	18	*		
Target Disability Type	14	*		
Prevention, Early Detection	8			

Evaluation & Monitoring	14	*	**PROCESS MANAGEMENT ISSUES**	**25**
Coverage Service Provision	14	*		
Efficiency & Effectiveness	8			
Needs Assessment	3			

Medical Rehab, Clinical, Therapy	14	*	**TECHNICAL CBR SKILLS & ISSUES**	**24**
Specific Technology or Skill	12	*		
Aids Appliances Modifications	7			
Orthopaedics Orthotics Prosthetics	5			
Psycho-social Support	5			

(Extent of data – Node draws from: * * *more than 75%, * *more than 50%, or *more than 25% of the evaluation reports)

As reflected in both Table 1 and Table 2, the 51 nodes were also separately ranked according to the frequency with which they were evident across the reports. It was reasoned that those themes which were identified in many reports (reflecting greater extent of data) should be given greater prominence in in-depth qualitative analysis than those which were only mentioned in a few reports. The criteria for classifying extent of data were:

**Table 2:** Nodes (themes) included/excluded – with descriptions.

**Included or Excluded**	**Node Title**	**Node Description**
**Included – Key Nodes: **Drawing from more than 75% of reports	Training	Recommendation that describes or relates to training, the need for training, processes for training, etc.
	Funding, Finances	Recommendation that pertains to finances, financial matters, funding and the management of funds.
	Collaborating Organisation	Recommendation pertains to a collaborating organisation. Mention of link with other organisation (has overlap with NGO node)

**Included – Major Nodes: **Drawing from less than 75% but more than 50% of reports	Management Issues	Recommendation relates to management functions, processes, activities and concerns, and the management of work related issues.
	Government – Links Agencies Dep'ts	Recommendation describes or pertains to government, government role or responsibility or relationship with government department or service or agency
	Expansion	Recommendation regarding expansion and expansion plans of the project. Increased services, new areas, new types of service.
	Community Involvement	Recommendation describes or pertains to community involvement with the project, and to work in local communities or villages. Includes community mobilisation. May have overlap with community development.
	NGO INGO	Recommendation pertaining to an NGO/INGO or relationship with an NGO/INGO. Often where NGO is provider of funding, of expatriate staff or expert input.

**Included – Minor Nodes: **Drawing from less than 50% but more than 45% of reports	Documentation	Recommendation describes or pertains to documentation in the project the collecting and use of data, surveys, documentation of activities, people etc., and reporting on these.
	Organisational Development	Recommendation regarding or pertaining to organisational development – usually of the project or its parent organisation. Resources for or training for or importance of organisational development.
	Income-Generation Vocational	Recommendation which pertains to income generation for people with disabilities, work-related, includes vocational issues.
	Education – integrated	Recommendations which describe or pertain to inclusive education, disabled children in schools, special schools, etc

**Included – (Summarised) Minor Nodes: **Drawing from less than 45% but more than 25% of reports	DPOs self help groups	Recommendation describes or pertains to any self help or DPO initiatives, either the fostering of them, the need to link with them, the need to dissociate from them.
	Participation in project	Recommendation describes or pertains to increasing participation of people with disabilities in the project (in terms of methods used, roles, etc) or participatory strategies that boost general participation of people with disabilities
	CBR worker LS, Role, Recruit	Recommendation describes or pertains to the role or position or work of the (usually paid) local supervisor or CBR worker. Includes recommendations to recruiting to this position
	Volunteers	Recommendation describes or pertains to volunteers, village level volunteers, volunteering in CBR and associated concepts.
	Planning	Recommendations which include statements about planning, the need for planning, the sort of planning that should be undertaken, etc.
	Policy	Recommendation pertains to: government policy, the need to influence policy; organisational policy, the need for, etc. The project's connection with policy.
	Family Involvement	Recommendation which pertains to the involvement of family members in the project, suggestions to increase or build family involvement.
	Medical Rehab, Clinical, Therapy	Recommendation that mentions medically oriented rehabilitation, physical or clinical therapies; including the provision of, need for, etc.
	Evaluation & Monitoring	Recommendation pertains to monitoring and evaluation – either in future, data that should be collected for, or current monitoring needs.
	Target Disability Type	Recommendation pertaining to specific type of disability, responding to needs of people with a particular type of disability. Resources or skills required for responding to the needs of people with a specific type of disability.
	Administrative	Recommendation describes or pertains to administrative issues or functions
	Coverage Service Provision	Recommendation describes or pertains to coverage of services, methods of service provision, improving service provision or coverage
	Staff Development	Recommendation relating to staff development in general (may have overlap with training)
	Specific Technology or Skill	Recommendation describes or pertains to a particular technology or skill that is required in a CBR project.
	Attitudes to people w/disability	Recommendation pertains to need to address attitudes to people with disability – either in society in general – or within the project.
	Grass-Roots Orientation	Recommendation which pertains to grass-roots, village-oriented or bottom-up approach. May have overlap with community involvement and decentralisation
	Research	Recommendation describes a research need, suggests a research topic or area, identifies research-related issue.
	PR and publicity	Recommendations pertain to the areas of public relations, improving public image and the public awareness of the project (sometimes overlapping public awareness of disability in general).

**Excluded: **Drawing from less than 25% of reports.	Empowerment	Recommendation pertaining to the empowerment of people with disabilities. Activities, training, etc which is for that purpose
	Efficiency & Effectiveness	Recommendation pertains to efficiency or effectiveness in a CBR project. The need for attention to, recommendations to increase and strategies for maximising efficiency and effectiveness
	Work Process, Teams	Recommendation describes or pertains to the process of work. The work processes within the CBR project.
	Prevention, Early Detection	Recommendation for or pertaining to (disability) prevention, early intervention, early detection type activities.
	Gender	Any gender-related recommendation. Recommendations which pertain to gender issues, gender equity, gender matters in the project.
	Advocacy	Recommendation describes or pertains to some form of advocacy or the need for advocacy. Usually individual advicacy
	Sustainability	Any reference to project or programme sustainability

**Excluded: **Drawing from less than 20% of reports.	Aids Appliances Modifications	Recommendation describes or pertains to any mobility or other aids or appliances, modifications to buildings or relevant equipment
	Decentralisation	Recommendation describes or pertains to the decentralisation of services. Moving services out of offices and into communities. Going to smaller villages or regional areas.
	Holistic Approach	Recommendations for or pertaining to a more holistic approach in the project.
	Orthopaedics Orth-otics Prosthetics	Recommendation pertains to provision of orthopaedic/orthotic/prosthetic appliances or assistance. The need for, training for, referral to.
	Psycho-social Support	Recommendation pertaining to psycho-social issues, psycho-social support and interventions.
	Human Rights	Recommendations that make mention of or pertain to human rights issues
	Village Committees	Recommendation pertains to village CBR committees (instigation, management, training, process, representation of, assistance for etc.)

**Excluded: **Drawing from less than 10% of reports.	Negatives, Descr-iption of Problem	Statement that describes a problem, negative situation, issue to be addressed.
	Needs Assessment	Recommendation describes or pertains to needs assessment. Recommendations for, about, suggestions on, etc.
	Deinstitutionalisation	Recommendation pertains to deinstitutionalisation – in the setting up of CBR or in institutional practices that have emerged in the project.
	Cultural Relevance	Recommendation pertains to local culture, attempts to respond to or be more relevant to local culture. Impact of culture on CBR
	Ethics Confidentiality	Recommendations for increased confidentiality or ethical practice. Pertains to some ethical issues in CBR practice
	Sexuality	Recommendation pertaining to sexuality and related issues
	Infrastructure	Recommendation relating to organisational or project infrastructure, infrastructure needed,

Key nodes (themes) – mentioned in more than 75% of reports.

Major nodes (themes) – found in more than 50% (but less than 75%) of evaluation reports.

Minor nodes (themes) – mentioned by more than 25% (but less than 50%) of evaluation reports.

Excluded nodes (themes) – those themes which emerged from the qualitative analysis, but which were mentioned by fewer than 25% of evaluation reports were not included in the current analysis (but are noted in Table 2).

## Results

The method adapted for this proof-of-concept study resulted in the identification of a broad range of informative themes. Specifically, at a methodological level, after conversion of all data to electronic text, the process was a straightforward coding task. Evaluation reports (and specifically recommendation sections) were readily amenable to thematic analysis. The themes were discernable from the text without undue extrapolation, and could be grouped conceptually.

At an informational level, 51 nodes or themes were identified from recommendation statements through the coding and analysis process. These nodes and their descriptions (Table 2), defined during the coding process to enhance accuracy and consistency of categorisation, were also used in subsequent reliability checking.

The conceptual grouping of these nodes, determined by consensus is depicted in Table 1. (The left columns show the spread of emphasis across the 51 nodes and reflect that each of the nodes or themes drew from between 1 and 30 of the 37 evaluation report recommendation sections). Likewise, the right side columns of Table 1 show the number of documents from which each of the broader conceptual categories drew. These columns indicate that the spread of categories was wide and that each category was evident in the majority of evaluation reports

**Figure 1:**
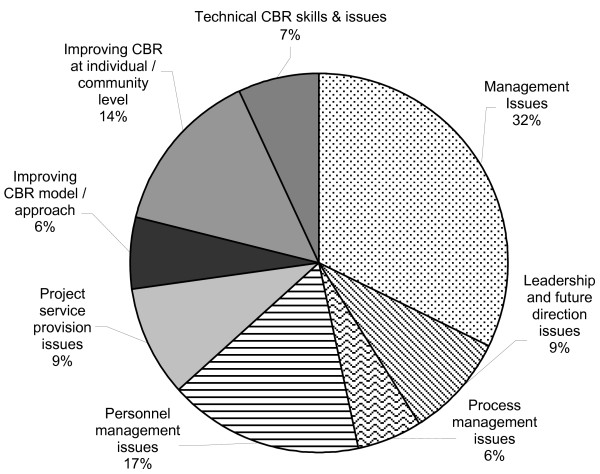
Percentage of passages coded across categories.

A parallel presentation of this data, indicating the relative strength of each of the major conceptual categories is presented in Figure 1. This figure, based on tallies of actual "passages" of data coded, rather than number of documents coded, shows relative percentages of data within each of the eight major categories.

In addition to the tables, Additional file 1 illustrates that recommendation sections of evaluations are a rich source for content and thematic analysis. The right column reflects the number of nodes or themes that were drawn from each of the evaluation report recommendation sections. The frequency ranged from 4 to 36 (out of 51) themes within each document, with a mean of 17.1. That is, on average 17.1 (30.5%) of the nodes or themes were apparent within each of the evaluation report recommendation sections.

### Qualitative analysis exploring key themes for CBR policy and planning

More detailed qualitative analysis, investigating each of the key concepts was also undertaken. These results, prioritised according to numerical strength (the number of times that concepts were mentioned in the recommendations) as well as the qualitative strength of the data (the context and potency with which concepts were conveyed), are presented below including representative quotes. They provide some descriptive understanding of the content of evaluators' recommendations. The descriptions also form the basis for a number of synthesis summary statements (underlined). These statements highlight the main issue within each theme, and are intended to inform managers, planners, implementers, policy makers and funders, based on the "evidence" of 37 evaluation report recommendations.

To ensure confidentiality of reports, all initially included reports were assigned random numbers and these are used to identify quotes, along with the general region (i.e. "Africa", "SE Asia", "South Asia", and "Other" regions).

### Key themes

The following three themes were the most strongly evident in recommendations across all CBR evaluation reports.

#### Training

On the basis of evaluation reports reviewed, training was the strongest theme. The training node comprised a number of small sub-themes (such as accreditation, certification, planning for training, continuing training and evaluating training outcomes). However, two major and multifaceted sub-themes pertained to (a) the intended recipients of training and (b) the recommended content of training.

##### a. Recipients of training

Recommendations of evaluators strongly emphasised that training should specifically be provided to project staff and related workers. There were many statements to the effect that "*there is a need ...for more advanced training for both local supervisors and CBR workers on regional and subregional levels*" (#9 Africa). Far fewer statements in this node related to training for people with disabilities, family members, volunteers and community members e.g. "*weekly sessions with people with disabilities, family members...and community leaders ...could offer training in key skills*" (#39 SE Asia). In summary, evaluation recommendations reflected a clear need to invest in staff training – primarily for CBR workers and professional staff.

##### b. Content of training

In keeping with the above, the primary recommendations regarding the content or type of training pertained to issues of concern to project staff and paid employees. Based on evaluation recommendations, the key content areas for training in order of importance were:

• rehabilitation-specific issues, e.g. "*skills that are critical *[for CBR workers] *are advanced skills and knowledge concerning different kinds of disabilities, vocational training, special education and adaptation of environment*" (#9 Africa),

• management-specific issues, e.g. "*provide management skills training to *[senior staff], *include planning, budget development, writing proposals and reports, supervision skills and directing personnel*" (#17, South Asia), and

• training in areas of concern to people with disabilities, family and community members, were also mentioned but with less emphasis.

In summary, evaluators' recommendations stressed that CBR training should emphasise rehabilitation-specific and management-specific skills training.

#### Funding, finances

The majority of evaluators, regardless of project type or country, made recommendations on funding and financial matters. Detailed analysis indicated a consistent sub-theme pertaining to financial processes, budgeting and financial accountability. Correct and transparent financial procedures were a major concern (nearly half of the recommendation statements within this node related to such issues). For example "*It is recommended that proper financial administration be set up and well maintained ... setting up better bookkeeping procedures ... whereby the issuing of money can be separated from other aspects of bookkeeping*" (#47 Africa). Local fundraising was also an issue that many evaluators recommended. In summary, correct and transparent financial and accounting procedures must be a key priority for CBR services and funders.

#### Collaborating organisations

Reference to collaborating organisations was common and strongly emphasised in CBR evaluation reports. Indeed the strength of these recommendations may reflect evaluators concerns over the insularity of CBR projects. The themes evident within the recommendations pertained to, the type of collaborating organisation (evaluators' recommendations related to local, government and international NGO sectors), and the reason for the recommended collaboration. Primary reasons for recommending greater collaboration included:

• skill development, e.g. "*links should be made to other professional development initiatives*" (#5, Other),

• linking and networking, e.g. "*list potential ... partners in government and NGO sectors ...for networking for mutual benefit *[to]*exchange information and resources*" (#24 South Asia), and

• funding related issues, e.g. "*the project needs to extend their liaison with other NGOs, government bodies and banks to increase the amount of funds available*" (#39, SE Asia).

In summary, CBR projects should more actively collaborate across local organisations, government departments and international NGOs to enhance skills, increase networks and to secure funding.

### Major themes for CBR policy and planning

The following five themes were highly important, judged on the frequency and the qualitative emphasis they were given across evaluation recommendations.

#### Management

As reflected in Table 1 and Figure 1, recommendations to improve management capacity comprised the key "meta-theme" in CBR evaluations. As might be expected within this specific node or theme, evaluators saw that "*management needs to be empowered with management, social and communication skills*" (#49 SE Asia). They identified the need for "*a proper management structure*" (#48 SE Asia) and improvement in "*business management*" (#35 Africa) and management of "*therapeutic and technical areas*" (#14 Other). However two other categories of recommendations were also evident, namely: the need for more participatory management skills or style, and the need for more strategic and visionary management and leadership. In summary, management weaknesses must be addressed, and recruitment, training and support for CBR managers should emphasise participatory styles, strategic leadership and vision.

#### Government – links, agencies, departments

Aligned with the "Collaborating organisation" node, this is another important theme that emerged from the data. Clearly CBR projects are often highly subject to, and connected with government agencies. Evaluators consistently recommended strengthening these connections even further, often to promote sustainability of CBR activities, but also to exert some constructive influence over government departments. The comment "*increase linkages with the health sector*" (#17 South Asia) is representative, as is "*the organisation ... needs to continue its catalyst role in strengthening and extending services ... of the state in district and rural areas*" (#20 South Asia). That is, evaluators also emphasised the potential influential role CBR projects might play with government departments, lobbying them at practical and strategic levels. In summary, CBR projects can not work independently of government agencies; they must build collaborative linkages where possible, and seek to influence government departments on disability and service issues.

#### Expansion

Understandably, the topic of service expansion was one of the major themes evident in the data. However, surprisingly the content of the recommendations coded to this node indicated that many evaluators were quite hesitant about project expansion. Half of the recommendation statements in this theme were generally cautious about expanding or extending project activities, e.g. "*it would seem wise not to implement in further *[districts] *until some clear strategies have been worked out with district partners, to work towards sustainability*" (#1 SE Asia). Interestingly four evaluations recommended against expansion and one recommended a reduction of services. With only a third of the relevant statements recommending actual expansion, it might be speculated that projects (and or donors) may at times have unrealistic expectations for the growth of CBR projects, which the majority of evaluators warned against. In summary, CBR project expansion plans or schedules are often unrealistic. Expansion should be guided by service realities, the availability of necessary staff and resources.

#### Community involvement

While often referred to by evaluators, there was no strong sub-theme evident across these statements. Recommendations related to the requirement for community involvement to be initiated, funded, and trained for. They described the need to enhance awareness, attitudes, mobilisation, and cultural issues, e.g. "*focus must be on assessing the existing local strengths and resources in a dialogue with the communities*" (#16 Africa). They also suggested "*private sector involvement*" (#42 South Asia) as a potential aspect. The synthesis did not indicate any means of enhancing community involvement. This is a potential area for research. In summary, community involvement is an important goal for CBR projects, but there appears to be little consensus as to how it can be achieved.

#### NGO, INGO

Recommendations arising from just over half of the coded reports comprised this theme. NGOs and INGOs were clearly seen as vital for CBR, e.g. " [CBR project] *needs to develop long term partnerships with local NGOs for the implementation of its programmes*" (#43 South Asia). Recommended roles of such organisations included providing technical expertise, funding and training as well as being a provider, collaborator, supervisor, and advocate. In general, recommendations suggested that greater links with NGOs and INGOs would result in enhanced implementation, support, resources, direction, learning and extension outcomes for CBR projects. Evaluators also warned on a few occasions of the "*damaging effects of short-term initiatives by NGOs*" (#22 Africa) and that NGOs should "*develop local competencies, not create dependency*" (#23 Africa). In summary, NGOs and INGOs are a key foundation for CBR services and a vital anchor and resource for projects, but at times caution may be advised.

### Minor themes for CBR policy and planning

#### Documentation

This node reflected numerous calls for clear, functional data and documentation pertaining to, (a) clients, (b) disability prevalence and surveys, (c) databases, and (d) financial and organisational data. Evaluators recognised that better data and documentation would improve the measurement of client needs and client outcomes, that it would "*show changes that reflect the impact of the work*" (#25 SE Asia), "*enhance planning and monitoring of service provision*" (#42 South Asia), improve "*planning decision making and improve programme performance*" (#6 SE Asia) as well as assist with accountability and report writing. In summary, clear guidelines and practical strategies on documentation and data collection, analysis, and usage are required in CBR.

#### Organisational development

Within recommendations for organisational development, the primary concern pertained to how such development might occur. The majority of recommendations related to plans and strategic planning, for example, " [CBR project] *should design a strategic development plan to ensure ...viability and sustainability*" (#2 SE Asia). Evaluators recommended an organisational development approach based on strong leadership, careful planning, which nurtures other organisations, and which is also reflective, promoting discussion and input. In summary, organisational development for CBR projects must include careful planning, sound management and internal and external collaboration.

#### Income-generation, vocational

This theme included mention of a broad range of issues including monitoring, training, procedural issues, skills, prioritisation, self employment, supported employment, open employment and training, loans and micro-credit. The need for greater understanding of this issue was evident in calls to *"look into alternative approaches" *(#9 SE Asia), and *"Study *[the] *income generation potential of different employment support mechanisms to allow customised regional plans" *(#17 South Asia). The inherent tensions between the disability service functions versus income-generation and vocational functions were clear, "*too much emphasis has been put on the group's role in income generation *[rather than] *members' education*" (#44 SE Asia). The most commonly mentioned sub-theme was loans and micro-credit, including the dilemma this activity raises for many CBR groups (e.g. conflict, problems with repayment, problems over interest charges, amounts of loans, and disbursement of loans). Our analysis indicates that this aspect requires careful attention on a global scale. In summary, income generation and vocational elements of CBR projects are a source of some tension. The place, benefits, and limitations of loans and micro-credit in CBR should be a priority area for the development (and/or application) of practical guidelines.

#### Integrated education

This theme reflects a strong emphasis on the need to substantially develop integrated/inclusive education (IE) in schools so that *"where possible, disabled children will be referred to local village schools" *(#7 Africa). The qualitative strength of comments in this theme indicates that this is an area where CBR projects may need to ensure greater emphasis and resources. In summary, CBR projects should seek to prioritise and support integrated education as a key part of their work.

#### Disabled person's organisations (DPOs)/self help groups

In CBR evaluations reviewed, the terms DPOs and self help groups were often used interchangeably. In some reports, both or one of these referred to formal, national organisations of people with disabilities and in others, these terms referred to village level advocacy groups. In general though, recommendations that referred to more national DPOs reflected tensions and uneasy relationships, for example "*the formation of ... DPOs ...has led to fragmentation and spread of resources*" (#8 Africa) with calls to clarify relationships more clearly. In the case of village level advocacy and self help groups, most recommendations pertained to the need for CBR projects to initiate, support, promote and formalise such groups " [CBR Project] *should promote different disabled people's or parent's groups ... informal organisations should be encouraged*" (#22 Africa). In summary, CBR projects should foster and work through village level self help groups, and clarify their relationships with independent (or linked) national organisations of people with a disability.

#### Participation in project

Maximising the participation of people with disabilities and their family members in CBR was also an important element to many recommendations. Analysis of this node indicates evaluators recommended two major means of maximising participation. Namely, (a) adopting a more participatory approach, "*there is a need to have a more inclusive or participatory management style. This is an urgent need*" (#4 Africa), and (b) addressing the role of people with disabilities, their involvement and their status in the project "*involve more disabled persons ... on an organisational level*" (#41 Other). Other strategies recommended for maximising the participation of people with disabilities were in: staff training, staff selection, and through supporting the formation of self help groups. In summary, greater participation of people with disabilities and family members in CBR could be achieved through more participatory organisational processes, and maximising the formal role of people with disabilities in projects.

For brevity, the remaining 16 minor themes and corresponding summary statements are presented in Table 3.

**Table 3:** Summaries of additional minor nodes

**Node (Theme)**	**Summary of findings**
CBR worker, local supervisor – role, recruitment	CBR workers are fundamental to CBR practice. In planning and policy making, priority should be given to ensuring CBR workers have adequate training, work conditions, incentives, role clarity and management support.
Volunteers	Volunteers need clear mechanisms and strategies for support in CBR Projects.
Planning	Clear and logical planning is very important for CBR.
Policy	Government and NGO policy impacts substantially on CBR practice, and therefore CBR projects and staff should be supported to inform and influence policy across respective agencies and departments.
Family involvement	Enhanced involvement of families in CBR will require a number of different approaches, including motivation, appropriate models, greater information and enhanced support.
Medical rehabilitation, clinical, therapy	Evaluations indicate that there is a need for debate and research on the place of, and the strategies used in, medical and clinical rehabilitation in CBR.
Evaluation and monitoring	The development (and/or dissemination) of evaluation and monitoring tools which measure effectiveness, outcomes and long term outcomes in an accessible, yet comprehensive manner must be prioritised.
Target disability type	A key issue for specialised focus in CBR is to assist people with intellectual disability.
Administrative	CBR practice would benefit from enhanced procedures and frameworks for client-related data management and financial administration.
Coverage, service provision	Expanding coverage in CBR requires cautious planning to balance quality and extent, as well as local community responsiveness.
Staff development	Staff development is an important investment into the sustainability of a project, for which careful planning is required.
Specific technology or skill	CBR training should include the need for disability-specific skill acquisition.
Attitudes to people with disabilities	CBR projects must seek social and community attitude change, primarily through training and awareness-raising.
Grass-roots orientation	CBR should seek a greater grass-roots orientation through family and child led processes, greater inclusion of local cultural factors, and through seeking alternatives to prescriptive outreach models.
Research	CBR needs applied, project level research, particularly with a community-wide focus.
Public relations & publicity	Public relations and publicity are important considerations for a CBR project.

## Discussion

With regard to our initial questions, we found that the analysis process was relatively straightforward, and resulted in meaningful and discernable themes. Independent generation of themes, cross checking between authors and involvement of experienced CBR professionals resulted in a rigorous and repeatable process and served to authenticate the results which were representative of, and grounded in the data.

### Limitations

A core goal of the current proof of concept study was to explore limitations of the data and process, and where possible to consider means of ameliorating these limitations. As such our discussion of limitations comprises a key aspect of the outcomes from the study. For example, some readers may note that evaluation reports may be misleading data sources because they often occur at the end of a project, and therefore they (and particularly their recommendations) may be solely for the benefit of funders, and for audit purposes [16], rather than to enhance the project or to inform CBR practice. Consequently, it was determined in the present study, that a criterion for inclusion was that a report should pertain to ongoing projects (as far as could be discerned from the report itself), to ensure that the intent of the report (and recommendations) would be to inform and improve CBR practice.

It may also be observed that a limitation of working with evaluation reports and recommendations might be that reviewers cannot take into account whether an evaluated project was successful or not beyond the period of the report. Projects may have gone on to improve (or fail, or change, or stagnate, or struggle) after an evaluation or even as a result of implementing the evaluator's recommendations. We believe that to some extent we have addressed this limitation by drawing a large number of reports (in this case 37 reports) from a variety of projects (36 projects) and evaluators (35 separate evaluation teams or individuals), from a variety of funders (in this case over 15 funding agencies) in 22 countries. We suggest that this diversity of data sources substantially compensates many such vagaries and anomalies in projects, implementation or evaluation.

Researchers will also recognise that recommendations are an imperfect data source because they typically focus on what can feasibly be done in a project, in a given period of time, often with limited resources – not what should be done (in an ideal world). We acknowledge this limitation, but also note that there is considerable benefit in basing a review to inform practice on "real world" rather than "ideal world" recommendations. Likewise, the focus of evaluation recommendations is typically on shortcomings of projects and emphasizes evaluator's suggestions for improving practice. As such, this or any similar synthesis is likely to be skewed towards issues that need to be addressed, rather than on aspects of CBR projects that are working effectively. While such constraints must be kept in mind, as recognised in the original exploration for this study [7], the inclusion of SWOT analyses in future syntheses will more effectively balance the bias towards 'weaknesses' and 'threats' with information on 'strengths' and 'opportunities'.

Another potential critique of the synthesis of recommendations (as in this proof-of-concept study) is that they are devoid of context. Without incorporation of the larger evaluation report (let alone an understanding of the project itself), this data may be vulnerable to misinterpretation or misrepresentation. This again is a valid concern. Without the inclusion of the full report, many nuances and contextual issues are lost, and we envisage that the full implementation of this methodology [7] would be less subject to such a limitation. However we also suggest that the process of extraction from the individual context and focusing on the issues, as they are described by the evaluators across a number of reports, has compensating benefits. It permits the analysis of multiple evaluators' concerns, taking them at face value. Indeed this approach may be particularly appropriate for a summarizing or synthesizing task, and is likely to elucidate a number of key issues that have wider relevance beyond a particular CBR project or setting.

A limitation that our review noted was that many evaluation reports are also "political" documents. That is, they are written for a specific audience (often a funder), they typically use tactful, measured language, sometimes make oblique recommendations, and seek to reconcile multiple agendas and stakeholders. Further, evaluations are not conducted to any formula, and may be critiqued as more accurately reflecting the perspective of the evaluator than the reality of the project being evaluated. A case in point here is the emphasis on management issues noted in the current project. It may be suggested that most evaluators have a strong interest in management issues; indeed many evaluators have substantial management background and experience. Additionally, this emphasis may be reflective of the Terms of Reference (ToR) under which evaluations are conducted. It is possible that the ToR set for evaluations by those who commission them reflect a bias towards management concerns. Such factors should be considered in any future study, and indeed ToR might form an integral comparative part of the data in such a study.

As noted earlier, in this review we relied on the mitigating influence of multiple reports from a variety of evaluators in different types of projects across many countries. Despite this, we found that aspects of our methodology (primarily those tallying frequencies of themes, etc) could potentially be quite influenced by evaluator's repetition of certain issues in the text. Likewise, for consistency and objectivity in our study we were not able to distinguish between evaluators' major and minor recommendations and weight accordingly, and in cases where multiple recommendations related to one theme, this led to the bias of multiple coded entries. In the current study we sought to address these concerns as far as possible by balancing the quantitative tallying of themes with more qualitative reporting. This proof-of-concept study however has also indicated that other measures can be taken in future applications of the methodology. These would include the extensive consultation with stakeholders originally envisaged [7], including ToR as data, and may extend to including other documents such as project proposals and interim reports in the analysis.

Finally, it is also important to convey the degree of confidence that we drew from our reading of multiple evaluation reports. From our reading of CBR evaluations, it appeared that few if any evaluators sought to be disingenuous or misleading. Most evaluators genuinely sought (at times under very difficult circumstances) to produce balanced and objective reports based on credible data, resulting in pertinent and meaningful recommendations. Notwithstanding the limitations noted above, we contend that data arising from such a starting point deserves careful attention.

### Findings

Regarding the actual findings of the study, these data provide a fascinating insight into CBR practice from the perspective of multiple evaluators across many projects, in 22 developing countries. The identified themes (Table 1) may serve as a springboard for further research and comparative evaluation. The list of themes may form a basis for the development of comparative tools for assessing and contrasting the relative emphasis in projects, as a checklist for evaluation reports or other documentation, or as a framework for planning.

In the descriptive overview, we have outlined themes and indicated their relative quantitative priority. We have noted 31 summary suggestions for policy makers and planners, which are underlined in the previous section and Table 3. We have tried to avoid the pitfalls of unconvincing and unsubstantiated qualitative data by ensuring the quantitative strength of our themes was measured, but also recognised that in this kind of research, simple counting of themes is not necessarily reflective of the qualitative strength of a theme, which is presented in the more detailed qualitative description. Despite these efforts, it is important to emphasise that this study is entirely based on evaluation reports and therefore largely reflective of the, priorities, perspectives and opinions of CBR evaluators.

From Table 1, it is clear that the emphasis in the data on broader management type issues is very strong. Indeed, four of the eight node categories (Management, Leadership and future direction, Process management, and Personnel management) are all broadly pertinent to management concerns, and these categories are referenced by more reports than the non-management node categories. Figure 1 further illustrates this finding. Based on all of the actual passages coded, rather than number of documents coded, this graph illustrates strikingly that the major categories which pertain to management (patterned slices) as listed above, together comprise 64% of the passages coded. That is, almost two-thirds of statements within the recommendation sections of our sample of 37 CBR evaluation reports were associated with management functions.

Preliminary indications from these data are that the key recommendations for change in CBR projects relate to management. General management-related concerns were emphasised in most detail and most frequently in evaluator's recommendations. Indeed based on these findings, it might be concluded that the major weaknesses in current CBR practice globally is management-related. Extrapolating from this finding, the key priority for any policy framework, planning or implementation strategy in CBR should be to prioritise technical, organisational, administrative, and personnel management, including strategic leadership issues.

Not surprisingly, recommendations pertaining broadly to improving CBR at the individual and/or community level were also important. It is to be expected that evaluators of CBR projects would focus on personal and local community issues such as community involvement, self help, participation, as well as families and attitudes. These issues are of direct impact to people with disabilities and were rightly emphasised.

Technical and procedural type themes pertaining to the core work of CBR projects (covered in "Project service provision" and "Technical CBR skills and issues" categories), together comprised only 16% of recommendation statement passages (Figure 1), which for example is less than the category of "Personnel management" issues. We might observe then, that these evaluators saw topics of training, staff development and staff recruitment as requiring greater attention via recommendations and therefore presumably more problematic for CBR projects than all of the issues covered under topics such as clinical rehabilitation, therapy, aids and appliances, psycho-social support, integrated education, income generation and prevention. This is a striking finding; assuming that the methodology has accurately reflected current CBR practice and concerns, and given that it is somewhat tangential to the emphasis within CBR manuals and texts [17-19], it is cause for further debate and research.

Interestingly, only six percent of the coded recommendations pertained to the category of "Improving the CBR model/approach" and the majority of the nodes that comprise this category only drew from less than 25% of the reports. At face value this finding indicates that CBR evaluators had little cause to make recommendations about issues such as ethics, human rights, empowerment and gender. However, the limited number of recommendations is probably more reflective of the remit of project evaluations and the level of focus of evaluation reports. For example, there may be limited value in recommending conceptual changes in the CBR model to a specific project. While the methodological aspects of this issue were discussed under 'limitations' (above), for present purposes it is noteworthy that the "CBR model/approach" was the category about which fewest recommendations were made.

### Qualitative discussion

Similar to the descriptive overview depicted in the tables, the qualitative data description also reflects the need for clear and visionary leadership, with greatly enhanced planning and training across CBR projects. The recommendations reviewed call for substantially improved management systems, processes and structures. They suggest that for CBR to advance, a stronger management and training foundation must be established.

Core issues that evaluators emphasised were the need for greater organisational infrastructure in CBR projects, an emphasis on staff support and administrative processes, and a more consolidated and organisationally coherent approach to CBR. There were also consistent recommendations for CBR to be more formalised and accountable.

The call for substantial investment in training was also evident. According to evaluators, there is clear necessity for CBR curricula to be enhanced at all staff and stakeholder levels. Given that CBR is an approach which is fundamentally based on training [20], it is somewhat surprising that almost 30 years later, evaluators consistently saw this as a neglected area globally. We acknowledge that this emphasis might be an artefact of evaluation report writing (to recommend training is usually a reasonable recommendation; constructive and achievable). However the emphasis is strong in the current study, and we have identified a number of areas in which evaluators maintain that it is clearly necessary.

Across the themes in this synthesis, there were repeated calls for more collaborative approaches at a number of levels, which corresponds with published examples of successful CBR projects [21]. Many evaluators expressed the need for CBR projects to be more connected with other organisations, with disabled person's groups, with government departments, with communities, and also with political and social agendas; again echoing calls in the literature [22]. There were numerous recommendations for CBR to be more participatory at an organisational and service level, and more inclusive of families, communities and people with disabilities. While recommendations for greater collaboration are well understood in the CBR world [23], this synthesis suggests that they may not be reflected in practice.

In contrast, evaluators questioned some of the traditionally fundamental tenets of CBR such as the place of medical and clinical rehabilitation and income generation activities. This does not appear to have been taken up in the literature. The need for critical research and investigation as well as greater strategic direction setting in CBR is evident from this synthesis. Recommendations made by evaluators on policy and planning, organisational change and service expansion indicate the need for a broader strategic vision.

The emphasis within this synthesis on funding and accountability is also important to note. While again, from a funder's perspective this may be a key consideration, the importance of this theme may also reflect something of a weakness in the implementation infrastructure in CBR. It may be that more attention is warranted on financial and accountability mechanisms.

Clearly there are many questions that could be asked about these data, and this method lends itself to much deeper investigation of specific themes than can be dealt with here. Comparisons might be made between CBR evaluations in different countries, or evaluations of projects using different approaches, or working with different populations.

Similarly, questions about the content of specific themes, the relative proportions of themes and the absence of certain themes from the list could be explored in much greater depth. For example, it would be interesting to explore why themes such as "Sustainability" or "Prevention and early detection" were relatively rarely mentioned in recommendations (Table 2). It is possible that such issues may not be relevant to the remit of evaluators. From another perspective, readers may also question whether issues such as "Empowerment", "Human rights" and "Gender" issues are actually satisfactorily addressed in CBR practice, that they were so rarely mentioned in recommendations (Table 2), or whether this also reflects the priorities of evaluators or those commissioning evaluations.

The exploration of such questions would provide important insights into CBR practice, the evaluation process and focus of evaluation in CBR. Such exploration would also inform discussion on the limitations of the data, some of which were noted in the accompanying paper. Most importantly though, the current proof-of-concept study indicates that the method reveals substantial informative data, and warrants further and full application, ideally with substantial stakeholder involvement.

## Conclusion

This study has striven to suggest a new approach to data synthesis and evidence in CBR. While primarily mapping out the major categories and themes, it has sought to interpret data accurately and meaningfully, as well as identify and address limitations constructively. The methodology was found to be practical, achievable and reliable. It has revealed some important implications in relation to policy and planning of CBR programmes. We conclude that the process of synthesising evaluative reports may be a strategic tool in efforts to build an evidence base to inform future development of CBR programmes globally. We suggest that qualitative systematic review of project evaluation reports is a fertile and meaningful area for future research. Further, we believe that the application of the full methodology proposed initially [7], particularly including the strongly collaborative dimensions, will add substantial value and veracity to the process and findings. Finally, we observe that this methodology may also have substantial application to other areas beyond CBR, particularly those within the area of international health and development, which are often characterised by a dearth of traditional "evidence" type data, but which may have rich qualitative data resources in the form of project implementation analyses and evaluation reports.

## Competing interests

The author(s) declare they have no competing interests.

## Authors' contributions

PK and SH conceived of the study. PK led the design, analysis and drafting. All authors were involved in acquisition of data, review of methodology and drafts. All authors read and approved the final manuscript.

## Pre-publication history

The pre-publication history for this paper can be accessed here:



## Supplementary Material

Additional file 1 Evaluation reports included in the review, with number of nodes referenced in each. Reference details for each evaluation report reviewed, noting country in which the evaluation took place, and the number of nodes (themes) identified within each report. Click here for file
