# Supplementary material for: Systematic synthesis of community-based rehabilitation (CBR) project evaluation reports for evidence-based policy: a proof-of-concept study
Source: BMC Int Health Hum Rights. 2008 Mar 6;8:3. doi: 10.1186/1472-698X-8-3 (PMC2294110; doi:10.1186/1472-698X-8-3)
Supplement: Additional file 1 — Evaluation reports included in the review, with number of nodes referenced in each. Reference details for each evaluation report reviewed, noting country in which the evaluation took place, and the number of nodes (themes) identified within each report. [file 1472-698X-8-3-S1.doc]

## Additional File 1 – Evaluation reports included in the review, with number of nodes referenced in each

| Evaluation Report | Country of Project | No. of nodes coded |
| --- | --- | --- |
| Abu Jaber M, Katanani H, Al-Kumash M, Al-Zghoul R, Ateyat M. Evaluating the community based rehabilitation (CBR) program: Integrating the disabled in local community centers. Jordan: The Community Based Rehabilitation Program; 1996. | Jordan | 4 |
| Associazione Italiana Amici di Raoul Follereau (AIFO). Mid term evaluation report: WATCH. Nepal: Women acting together for change (WATCH); 2005. | Nepal | 17 |
| Boyce W. Mid term performance evaluation: Comprehensive Disabled Afghans Programme (CDAP). Afghanistan: UNDP; 1998. | Afghanistan | 30 |
| Chalker P. Evaluation report: Disability programme, Ho Chi Minh City. Vietnam: Save the Children, Vietnam; 1998. | Vietnam | 12 |
| Chidyausiki SR, Davies MP, Scherbaum H, Sidile ML, Mpunzwana PH, Zinkin P. Evaluation report of the Jairos Jiri Association activities. Zimbabwe: Jairos Jiri Association; 1996. | Zimbabwe | 27 |
| Claussen J, Kandyomunda B, Jareg P. Evaluation of the community based rehabilitation programme in Tororo District. Uganda: Community Based Rehabilitation Programme; 2005. | Uganda | 16 |
| Cornielje H. Appraisal: Pusat Rehabilitasi Yakkum, Indonesia. Alphen aan den Rijn, NL: Enablement; 2001. | Indonesia. | 24 |
| Cornielje H. Imani vocational training centre for handicapped children, Mtakuja, Kilimanjaro District, Tanzania. Alphen aan den Rijn, NL: Enablement; 2002. | Tanzania | 18 |
| Cornielje H. Report consultancy Sabatu, Rehabilitation of people with a handicap, West Kalimantan, Indonesia. Alphen aan den Rijn, NL: Enablement; 2004. | Indonesia | 14 |
| Danish Bilharzias Laboratory (DBL). Disability in Bangladesh: A situation analysis. Washington, DC: World Bank; 2004. | Bangladesh | 20 |
| Delneuville A, Bonnet J, Roath L, Jacobs H, Dos S. Capacity building of people with disability in the community (CABDIC). Cambodia: Handicap International; 2000. | Cambodia | 31 |
| Du Toit M. Evaluation of the Lesotho National Federation of Organisations of Disabled Persons - Development Activists Training Programme. London, UK: Save the Children, UK; 1996. | Lesotho | 13 |
| Evans P, Zinkin P, Chaudhury G, Venkatesh B, Harpham T, Chander SJ, et al. India urban community based rehabilitation project. India: Urban Community Based Rehabilitation (UCBR) Project; 1998. | India | 21 |
| Grut L, Hjort P, Eide AE. More of the same and try something new: Evaluation of the Community Based Rehabilitation Programme in Eritrea. Eritrea: The Community Based Rehabilitation Programme; 2004. | Eritrea | 18 |
| Harknett S, Bouamidi S, Bounleang P, Inthavong S, Khum K, Navajongpun C. Salavan DIGNITY project evaluation report. Lao PDR: World Concern Laos; 2002. | Lao PDR | 19 |
| Hoffmans T, DeRoos V. A decade of hopeful steps in Guyana: A participatory comprehensive evaluation of the CBR programme. Guyana: Hopeful Steps; 1996. | Guyana | 19 |
| Horvath R, Condor J. Disability Action Council: Assessment Report. Cambodia: Disability Action Council; 2001. | Cambodia | 8 |
| Kane TT. Disability in Vietnam: A meta-analysis of USAID associated general projects and data. Washington, DC: USAID; 1999. | Vietnam | 10 |
| Kaul S, Chhetri MB, Crishna B, Banerjee R, Sen R, Hamblin T, et al. SSEI and DfID Evaluation. India: Spastics Society of Eastern India; 1999. | India | 17 |
| Medi E, Dupret A, Fonseca W, Monroy W. National programme of Community Based Rehabilitation in Cape Verde: A participatory review. Cape Verde: NPCBR; 1996. | Cape Verde | 13 |
| Mendis P. CBR Program West Bank and Gaza Strip: Report of an evaluation mission. Oslo: Diakonia and NAD; 1996 | Palestine | 13 |
| Ministry of Labour Health and Welfare (MLHW). Evaluation of the CBR programme in Debub region: Tasting the fruits of the CBR programme. Asmara, Eritrea: MLHW; 1998. | Eritrea | 36 |
| Nangati F. Evaluation report of the Community Based Rehabilitation programme. Harare, Zimbabwe: Jairos Jiri Association; 1999. | Zimbabwe | 18 |
| Nordic Consulting Group. Review of the Community Based Rehabilitation Programme. Uganda: The Community Based Rehabilitation Programme; 2000. | Uganda | 14 |
| O'Toole B. Evaluation: Sulawesi Community Based Rehabilitation Programme. Indonesia: Sulawesi Community Based Rehabilitation Programme; 1998. | Indonesia | 20 |
| O'Toole B, Katende P, Kisubi M, Mulya D, Mpagi V, Nganwa A, et al. Participatory evaluation: Community Based Rehabilitation Programme. Uganda: The Community Based Rehabilitation Programme; 1996 | Uganda | 26 |
| Paul D. Peer Counselling Project: A partnership project between Social Services Cambodia and the Council of Canadians with Disabilities. Cambodia: Social Services Cambodia; 2000. | Cambodia | 9 |
| Payne H, Simwanza F. Mid term review report: Chipata District Community Based Rehabilitation Programme. Zambia: Chipata District - Community Based Rehabilitation Programme; 1988. | Zambia | 14 |
| Pierdomenico L. Report of activities: Community-Based Rehabilitation Programme. Vietnam: AIFO CBR Programme; 2003. | Vietnam | 10 |
| Rouse M, Florian L, Connolly J. External evaluation: Special Classrooms for Children with Disabilities, 1997- 2000. Bosnia and Herzegovina: Special Classrooms for Children with Disabilities; 2000. | Bosnia and Herzegovina | 11 |
| Stanggren J. Inclusive education in Vietnam: An evaluation of the support by Radda Barnen. Stockholm: Radda Barnen; 1996. | Vietnam | 5 |
| Stills M, Dunleavy K. Veterans International technical and medical rehabilitation support services in Cambodia. Phnom Penh: Veterans International; 2001. | Cambodia | 14 |
| Thomas M, Thompson S, Pahan S, Lakra V, Talukder PK, Marandi R, et al. Lamb Hospital Disability Programme, Rajabashor Parbatipur, Dinajpur, Bangladesh: Review and Strategy Planning. Bangladesh: Lamb Hospital; 2001. | Bangladesh | 25 |
| Warui P. A report on the assessment of Koscobar-K partner groups - undertaken in Korogocho Slum, Nairobi. Kenya: Partner Groups in Korogocho Slum; 2005. | Kenya | 9 |
| Werner D. External evaluation of the Community Based Rehabilitation (CBR) pilot project in Granma, Cuba. Brussels: Handicap International Belgium; 2004. | Cuba | 20 |
| Ziesler M. Impact assessment: Rehabilitation program in Palestine. Palestine: The Community Based Rehabilitation Progam; 2001. | Palestine | 14 |
| Zinkin P, Cassimo F, Colaco JC. Evaluation of Community-based Programme for People with Disabilities. Mozambique: Ministry for Co-ordination of Social Action; 1999. | Mozambique | 25 |
